# Minicells as an *Escherichia coli* mechanism for the accumulation and disposal of fluorescent cadmium sulphide nanoparticles

**DOI:** 10.1186/s12951-024-02348-0

**Published:** 2024-02-27

**Authors:** Felipe Valenzuela-Ibaceta, Nicolás Torres-Olea, Javiera Ramos-Zúñiga, Claudio Dietz-Vargas, Claudio A. Navarro, José M. Pérez-Donoso

**Affiliations:** BioNanotechnology and Microbiology Laboratory, Center for Bioinformatics and Integrative Biology (CBIB), Facultad de Ciencias de la Vida, Universidad Andrés Bello, Av. República # 330, Santiago, Chile

**Keywords:** Minicells, Quantum dots, Nanoparticles, Cell división, Cadmium, *Escherichia coli*, Tolerance mechanisms

## Abstract

**Background:**

Bacterial biosynthesis of fluorescent nanoparticles or quantum dots (QDs) has emerged as a unique mechanism for heavy metal tolerance. However, the physiological pathways governing the removal of QDs from bacterial cells remains elusive. This study investigates the role of minicells, previously identified as a means of eliminating damaged proteins and enhancing bacterial resistance to stress. Building on our prior work, which unveiled the formation of minicells during cadmium QDs biosynthesis in *Escherichia coli*, we hypothesize that minicells serve as a mechanism for the accumulation and detoxification of QDs in bacterial cells.

**Results:**

Intracellular biosynthesis of CdS QDs was performed in *E. coli* mutants Δ*minC* and Δ*minCDE*, known for their minicell-producing capabilities. Fluorescence microscopy analysis demonstrated that the generated minicells exhibited fluorescence emission, indicative of QD loading. Transmission electron microscopy (TEM) confirmed the presence of nanoparticles in minicells, while energy dispersive spectroscopy (EDS) revealed the coexistence of cadmium and sulfur. Cadmium quantification through flame atomic absorption spectrometry (FAAS) demonstrated that minicells accumulated a higher cadmium content compared to rod cells. Moreover, fluorescence intensity analysis suggested that minicells accumulated a greater quantity of fluorescent nanoparticles, underscoring their efficacy in QD removal. Biosynthesis dynamics in minicell-producing strains indicated that biosynthesized QDs maintained high fluorescence intensity even during prolonged biosynthesis times, suggesting continuous QD clearance in minicells.

**Conclusions:**

These findings support a model wherein *E. coli* utilizes minicells for the accumulation and removal of nanoparticles, highlighting their physiological role in eliminating harmful elements and maintaining cellular fitness. Additionally, this biosynthesis system presents an opportunity for generating minicell-coated nanoparticles with enhanced biocompatibility for diverse applications.

**Graphical Abstract:**

**Figure:**
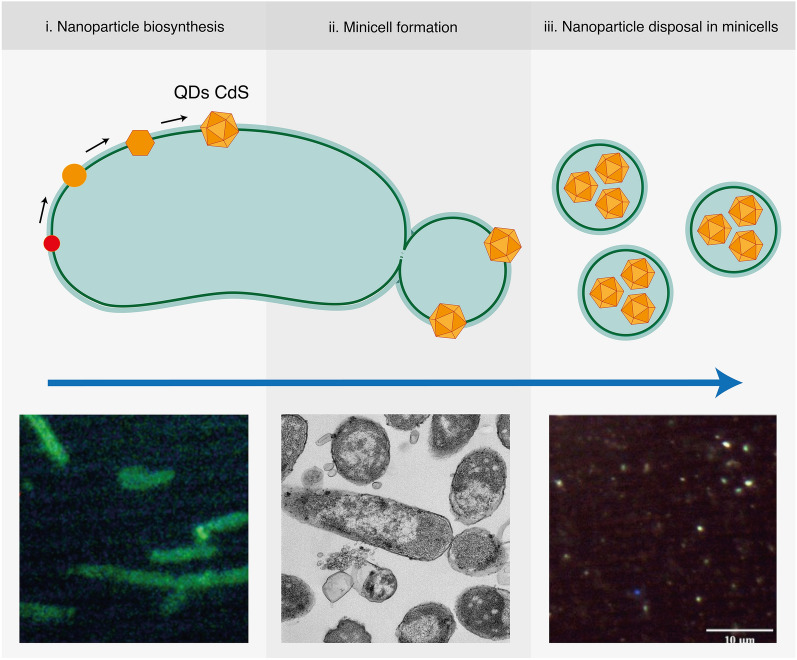

**Supplementary Information:**

The online version contains supplementary material available at 10.1186/s12951-024-02348-0.

## Introduction

In the realm of nanotechnology, recent years have witnessed remarkable advancements reshaping diverse fields, from electronics to medicine [1]. Quantum dots (QDs), a class of semiconducting fluorescent nanoparticles are particularly noteworthy for their exceptional optical and electronic properties, making them highly coveted for various applications, including bioimaging [2], biomedical uses [3], and optoelectronics [4]. Typically ranging in size from 1 to 10 nm, these structures comprise elements such as cadmium, copper, lead, zinc, tellurium, sulfur, among others [5]. The optical properties of QDs are governed by the quantum confinement effect, intricately tied to their size and elemental composition [6]. While conventional chemical methods continue to dominate commercial QD production, the utilization of biological systems has recently gained significant attention, owing to the heightened efficiency of the synthesis process [7]. Notably, microbial systems, such as *Escherichia coli*, a well-studied bacterium with versatile genetic engineering capabilities, offer a promising avenue for exploring various aspects of QD biosynthesis.

The majority of extensively studied QDs are comprised of heavy metals including cadmium, lead, or copper, many of which are toxic to biological systems. Within microorganisms, exposure to heavy metals can disrupt membranes and adversely impact various protein functions [8]. For example, cadmium toxicity in bacterial cells primarily stems from the metal’s binding to sulfur [9], leading to detrimental effects such as reduction in total glutathione (GSH) content [10], superoxide or other reactive oxygen species (ROS) formation [11–13], hypermutability due to inhibition of mismatch repair systems [14], replacement of essential divalent metals in enzymes that use these cofactor metals [15] and overall protein malfunction manifested through protein misfolding and the formation of inclusion bodies [16].

The proposed hypothesis by our and other research groups is that the biosynthesis of metal-based quantum dots (QDs) serves as a mechanism for bacteria to cope with heavy metal-related stress by facilitating the accumulation and immobilization of metal ions [17–20]. While a comprehensive understanding of this process remains elusive, significant strides have been made, particularly in the context of cadmium sulphide quantum dots (CdS QDs), to unravel how bacteria orchestrate QD formation through diverse metabolic pathways. For instance, CdS QDs biosynthesis is stimulated in *E. coli* and *Acidithiobacillus thiooxidans* when exposed to elevated phosphate concentrations [19, 21]. This phenomenon is attributed to the enhancement of cadmium uptake facilitated by the phosphate inorganic transport system (Pit), forming a metal-phosphate complex [22]. Additionally, molecules and intermediates involved in sulfur metabolism, such as GSH [23, 24], or volatile species like hydrogen sulphide or methanethiol [17], play crucial roles in the formation of CdS QDs. Furthermore, QDs synthesized by bacteria are enveloped by an organic layer composed of proteins and other biomolecules. This organic coating not only stabilizes the nanostructure but also mitigates their toxicity [25]. Of note, an underexplored aspect in QD biosynthesis is whether mechanisms exist for the elimination of these nanoparticles from the bacterial cell.

Previously, our research group documented the biosynthesis of cadmium QDs in a genetically modified strain of *E. coli*. In this system, the fluorescence emission from the QDs concentrates within defined cellular structures, discreetly positioned at cell poles or in the extracellular environment [23]. This outcome suggested that cell membrane stress, fragmentation, or even non-canonical division processes play a role in bacterial nanoparticle biosynthesis. Specifically, minicell formation, a form of polar non-canonical cell division, emerges as a plausible strategy for the cell to eliminate these biomineralized heavy metals, predominantly localized at its poles. Minicells are spherical cell structures resulting from polar division, devoid of chromosomal DNA, and consequently, they do not undergo division themselves [26]. Their generation occurs in strains with mutations in the Min division system, encoded by the *minC, minD and minE* genes [27–29], which regulate the proper division at the cell center, and when mutated, generate highly filamentous cells that produce minicells constitutively [30]. Although minicells have been extensively studied for their utility in biotechnological applications, such as simplified systems for studying membrane-level morphology [31, 32], their high biocompatibility, low production cost and non-pathogenic nature have positioned them as promising candidates for novel drug carriers [33].

The formation of minicells, initially considered to lack a physiological role due to the Min system deletion, has recently been shown to be associated with enhanced tolerance to stressors. Notably, research by Rang et al. [34] demonstrated that an *E. coli* Δ*minC* strain exhibited increased resistance to streptomycin, as the minicells generated in this strain facilitated the elimination of inclusion bodies and misfolded proteins induced by antibiotic exposure [34]. This finding holds particular significance as the misfolded protein stress induced by cadmium exposure, a requirement for QD biosynthesis, is akin to the stress caused by streptomycin [9, 16, 35]. Given that the primary localization of nanoparticles occurs at cell poles, we hypothesize that minicells may serve as a mechanism for the disposal of nanoparticles from *E. coli* cells.

This study investigates minicell formation as a potential mechanism for the disposal of CdS nanoparticles in *E. coli*. Biosynthesis of CdS QDs was conducted in minicell-producing strains, revealing the elimination of fluorescent nanoparticles from the cell within minicells. Importantly, this process significantly influences the optical properties of the synthesized nanoparticles. To the best of our knowledge, this marks the first report elucidating the relationship between minicells and the elimination of metal nanoparticles from bacterial cells.

## Materials and methods

### Bacterial strains

Mutant *E. coli* strains Δ*minC* and Δ*minCDE* were derived from the parental strain BW25113, using standard λ-red recombineering [36]. Primers used are shown in Table 1. Strains were routinely grown in lysogeny broth (LB; composition: 0.5% yeast extract, 1% tryptone, 1% NaCl). Strains were kept in LB plates (2% agar), with 25 µg/ml chloramphenicol for mutant strains.

**Table 1:** Primers used in this study

Name	Sequence (5’–3’)	Construction/purpose
minC-P1	GCAGAACCTAAGGTTATCCATCAGGCGCTGGAAGACAAAATCGCTGTGTAGGCTGGAGCTGCTTC	Δ*minC*::*Cm* and Δ*minCDE*::*Cm*
minC-P2	CGACTAACTGCAGTCGCGCCGCTTTGCCATAAAATTCTGCTGGGAATGGGAATTAGCCATGGTCC	Δ*minC*::*Cm*
minCDE-P2	AAGAATAGAAATATCGCCATCTTTTTGCTCAAGCTGTACGGTTACATGGGAATTAGCCATGGTCC	Δ*minCDE*::*Cm*

### CdS QDs biosynthesis

QDs biosynthesis was performed according to Venegas et al. [19] with modifications. A single colony of each strain was grown independently overnight in LB broth at 37 °C under constant agitation. Optical density at 600 nm of cultures was adjusted to 0.5 with fresh medium and a 100-fold dilution was prepared in M9-glucose minimal medium (composition: 42.2 mM Na_2_HPO_4_, 22 mM KH_2_PO_4_, 8.56 mM NaCl, 18.7 mM NH_4_Cl, 1 mM MgSO_4_, 0.1 mM CaCl_2_ and 0.2% glucose) and supplemented with 60 μg/ml of CdCl_2_. Cultures were incubated aerobically at different times at 37 °C with constant agitation. Cells were collected by centrifugation (7690 × g/30 min/4 °C) and washed with potassium phosphate buffer (50 mM/pH 7.4). To evaluate the presence of fluorescent nanoparticles, pellets were exposed to UV light (365 nm) for photographic record. As negative biosynthesis control, cultures with no addition of CdCl_2_ were prepared. For relative fluorescence intensity quantification, pixel intensity was measured using Fiji-ImageJ [37] from pellet photographs and normalized by the pellet wet weight.

### Fluorescence microscopy

For microscopic analysis, an epifluorescence microscope MF606 (BW OPTICS) was used. Bacterial pellets were resuspended in potassium phosphate buffer (200 μl/50 mM/pH 7.4). An aliquot of 5 μl was mounted on a glass slide without fixation. The samples were observed through 40X (NA: 0.65) and 100X (NA: 1.25) objectives. For fluorescence microscopy, samples were excited at 330–380 nm. Image analyses were carried out in the software Fiji-ImageJ.

### Transmission electron microscopy (TEM) and energy-dispersive X-ray spectroscopy (EDS)

Cells were collected by centrifugation after exposure to biosynthesis conditions. Obtained pellets were fixed in glutaraldehyde 2.5%, treated with osmium tetroxide 1%, and infiltrated with epoxy resin. Thin slides were obtained from fixed pellets using an ultramicrotome. Pellet slides were placed on a commercial copper grid. TEM micrographs were captured with a Talos F200C G2 microscope (Thermo Fisher Scientific), operated at 200 kV. For EDS study, the same grids were analyzed using an INSPECT‐F50 scanning electron microscope (Thermo Fisher Scientific), equipped with an EDS detector, operated at 20 kV.

### Minicells and rod cells enrichment

Minicells and rod cells from mutant strains Δ*minC* and Δ*minCDE* were partially purified using the methods described by Lai et al. [38] and Jivrajani et al. [39], with modifications (Fig. 5A). Pellets of mutant strains obtained from 200 ml cultures were concentrated and resuspended in 40 ml of LB broth. Bacterial suspensions were slow-centrifuged (1000 × g/10 min/4 °C). The pellet obtained by this step was enriched in whole cells, and the supernatant obtained was enriched in minicells. To further purify the minicell fraction, the supernatant was separated and incubated at 37 °C for 45 min with continuous agitation. Then, 150 μg/ml of ampicillin was added to the culture, and it was further incubated at 37 °C for 2 h. The culture was centrifuged (400 × g/5 min/4 °C) to eliminate cellular debris, until no pellet was observed. Finally, the minicell fraction was centrifuged (7690 × g/30 min/4 °C) and washed with potassium phosphate buffer (50 mM/pH 7.4). To increase the purity of rod cells, the enriched pellet was resuspended in 40 ml of potassium phosphate buffer (50 mM/pH 7.4), and centrifuged (1000 × g/10 min/4 °C). Then, the supernatant was discarded. This process was repeated a second time. Purity of obtained fractions was evaluated by optical microscopy.

### Nanoparticle content of minicells and rod cells

Fluorescence emission analysis was performed by pixel intensity, as described by Tian et al. [40]. Purified minicell and rod cell fractions were prepared for microscopy analysis as described above and fluorescence images were captured and analyzed using the Fiji-ImageJ software. Cells/minicells were randomly selected (n = 30) and total pixel density was calculated and normalized by the cell area. Cadmium quantification was carried out by flame atomic absorption spectrometry (FAAS). Enriched fractions were lyophilized, weighted, and digested in a microwave digestor, for which 0.05 g of dry lyophilized samples were placed on Teflon glass with 5 ml of nitric acid 60% and 4 ml of H_2_O_2_. Digested samples were filtered in a Whatmann N°42 filter and completed to 25 ml with distilled water. Samples were quantified on an atomic absorption spectrophotometer Perkin Elmer 3110, measuring absorption at 228.8 nm, slit 0.7 nm. Absorption data were compared with a calibration curve in a 0.028—2 mg/L range made using a cadmium 1000 mg/L Cd Certipur^®^ standard. To normalize the amount of cadmium by the volume of a single cell, the mean weight of a rod cell was estimated to be 1 pg [41]. Then, the representative length and width of rod cells was measured from 30 rod cells from each mutant strain using Fiji-ImageJ. The representative diameter of minicells was measured the same way. Based on the estimated weight and size, an estimated weight of minicells was calculated. To calculate the mean volume of rod cells and minicells, it was assumed that rod cells were cylinders and minicells were spheres. The dry weight of each sample was used to estimate the number of cells for normalization. Statistical analyses of two groups were performed by an unpaired two-sided t-test, using the GraphPad Prism software, considering as significant a p < 0.05.

### Sulphide detection assay

The production of H_2_S from minicells and rod cells was evaluated as described previously [42] with modifications. Δ*minC* and Δ*minCDE* strains were grown in LB liquid medium until OD ~ 0.5 and minicells and rod cells fractions were prepared as described above. The fractions were washed with sterile NaCl (1% w/v) solution and then resuspended in the same solution, to an OD value of 1. Aliquots of 5 ml of fractions were distributed in tubes and supplemented with 1 mM cysteine. A piece of filter paper soaked with 40 μl of a lead acetate solution (100 mM) was attached under the cap of each tube. Tubes were incubated at 37 °C for 24 h. Positive or negative reactions were recorded according to the apparition of a dark precipitate in the paper placed under the cap. Control reactions were performed in the absence of cysteine or cells.

### Nanoparticle purification

Cell pellets obtained from 100 ml biosynthesis cultures were resuspended in 10 ml of a 1 M NaOH solution and then incubated at 90 °C for 10 min. Pellets were recovered by centrifugation and then resuspended in 10 ml of 50 mM Tris–HCl pH 8.5 buffer with 1% SDS, and then disrupted by sonication. Cell debris was removed by centrifugation and filtration with a 0.22 µm filter. Nanoparticles were concentrated in 3 kDa Amicon filters (Millipore) to an approximate volume of 200 µl. These solutions were used for optical properties analysis in a Synergy H1 microplate reader (BioTek Instrument Inc.). Absorbance spectra were measured between 300 and 700 nm. Fluorescence spectra were measured between 520 and 700 nm, with a 360 nm excitation.

### Cytotoxicity analysis of CdS nanoparticles

To assess the impact of CdS nanoparticles on the wild type BW25113 strain, growth curves of *E. coli* BW25113 were conducted, evaluated as described by Helbig et al. [10], with modifications. An overnight *E. coli* culture was diluted 1:100 in fresh LB medium and incubated at 37 °C. After 2 h, it was further diluted 1:100 in LB medium supplemented with CdS nanoparticles (biological and chemical) and incubated until reaching the stationary growth phase. Concentrations of 250, 500, 750, 1000, 2000 and 3000 µg/mL were employed for each type of nanoparticle. Growth was monitored by optical density at 600 nm using a Synergy H1 microplate reader.

Chemical nanoparticle synthesis was carried out by the procedure previously described by Venegas et al. [19], with modifications. Reactions involved 200 mM CdCl_2_, 100 mM phosphate buffer, and 5 mM reduced L-glutathione (Sigma Aldrich), dissolved in borax-citrate buffer at pH 9.4. The reaction mixture was then incubated for 30 min at 90 °C. To eliminate any remaining metallic salts and synthesis reagents, the reactions were filtered using 3 kDa Amicon filters. Biological synthesis of CdS nanoparticles was carried out as described previously [4, 42].

## Results

### CdS nanoparticles are encapsulated in minicells

**Fig. 1:**
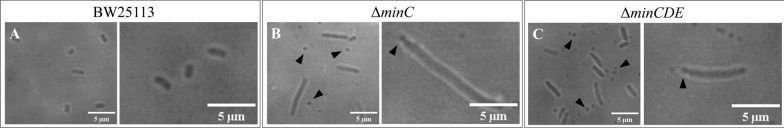
Minicell formation in Min deficient mutants. Representative optical microscopy images of overnight cultures of *E. coli* strains BW25113 (**A**), Δ*minC* (**B**) and Δ*minCDE* (**C**). Black arrows show minicells produced by the indicated strain

We chose to work with two well-studied mutations in the Min system for minicell production: Δ*minC* and Δ*minCDE*. Previous studies have reported that cells with a deletion in the *minC* gene (Δ*minC*) exhibit increased tolerance to cell stress [26, 34]. Conversely, cells with a deletion in the entire *minCDE* operon (Δ*minCDE*) have been widely employed as a source of minicells for morphological studies [31, 43]. To confirm the induction of minicell formation in Min-deficient mutants, overnight cultures were observed using optical microscopy. The parental strain BW25113 maintains the typical rod-like shape, with cells approximately 2 μm in length (Fig. 1A). In contrast, cells from the Δ*minC* and Δ*minCDE* strains exhibit a highly filamentous morphology, with numerous spherical minicells present in the field or being generated from cell poles (Fig. 1B, C, indicated by black arrows). This observation validates that the chosen mutations lead to the constitutive formation of minicells.

**Fig. 2:**
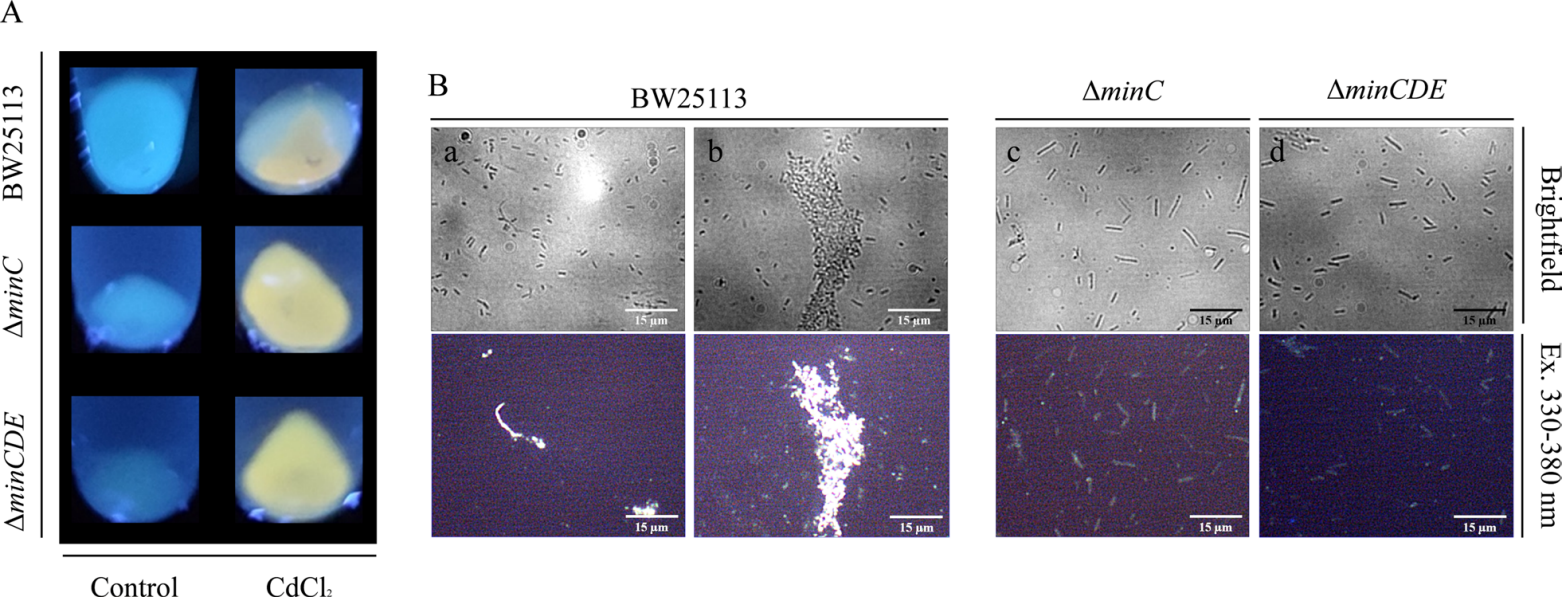
CdS intracellular biosynthesis in *E. coli*. **A** Bacterial pellets exposed to UV light (365 nm) of indicated *E. coli* strains grown on M9-glucose medium supplemented with 60 μg/ml CdCl_2_ or without metal (control) after 14 h at 37 °C. **B** Representative fluorescence microscopy images of cells from metal-exposed pellets after 14 h at 37 °C of BW25113 strain (a, b), Δ*minC* (c) and Δ*minCDE* (d). Fluorescence images were captured after excitation with a 330–380 nm filter

The intracellular biosynthesis of CdS nanoparticles was conducted by cultivating the strains in M9-glucose medium supplemented with CdCl_2_ during 14 h at 37 °C. The phosphate molecules present in M9 medium facilitate the intracellular biosynthesis of nanoparticles by promoting cadmium uptake via the phosphate inorganic transport system (Pit) and H_2_S formation in cells [19, 22]. Due to the intrinsic fluorescence of CdS QDs, their synthesis was assessed by exposing cell pellets to UV light. Previous reports suggest that the concentration of CdCl_2_ used can influence the fluorescence intensity of cells following biosynthesis conditions [40, 44]. We tested two concentrations of CdCl_2_ based on biosynthesis systems previously reported by our group: 10 μg/ml [23, 44] and 60 μg/ml [19]. With the supplementation of 10 μg/ml, only a faint fluorescence emission from the pellets was detected (Additional file 1: Figure S1). In contrast, the addition of 60 μg/ml CdCl_2_ to the growth medium resulted in noticeable fluorescence emission from cell pellets, indicating the synthesis of CdS nanoparticles by all three strains (Fig. 2A). Cell morphology and fluorescence emission were further analyzed using fluorescence microscopy. The BW25113 strain exhibited high-intensity fluorescence from cell aggregates (Fig. 2Ba, Bb). Our group previously investigated this behavior, attributing it to an increase in the formation of extracellular polymeric substances (EPS) under biosynthesis conditions as a mechanism for metal disposal from the cells (unpublished results). In contrast, cells from the mutant strains primarily exhibited fluorescence emission from individual rod cells (Fig. 2Bc, Bd), although some cells were observed forming fluorescent cell aggregates (Additional file 1: Figure S2). Microscopy observation of control cultures without the addition of CdCl_2_ revealed no fluorescence emission (Additional file 1: Figure S3).

**Fig. 3:**
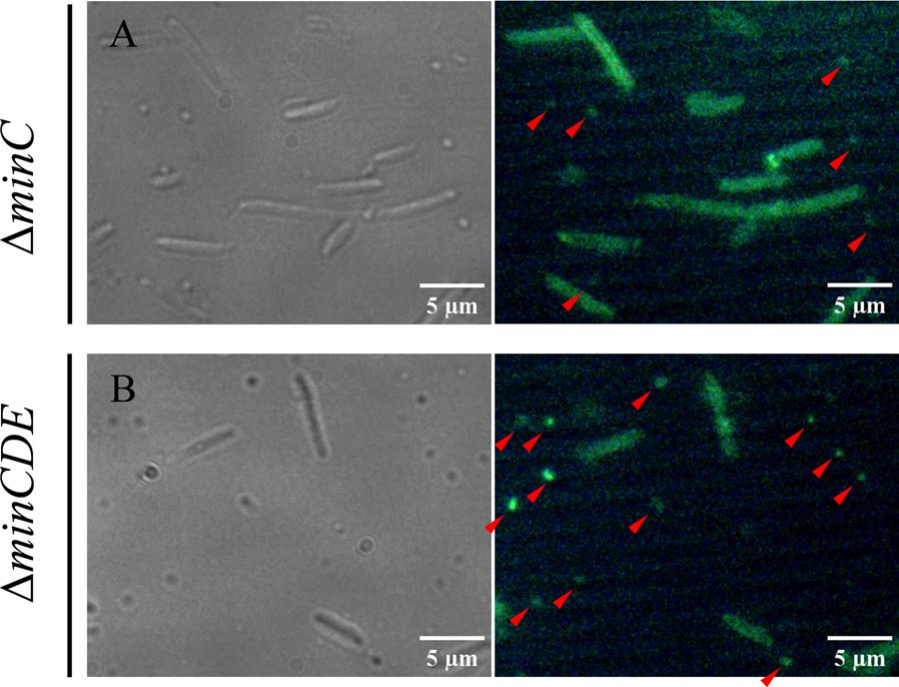
Fluorescence emission in minicells. Representative microscopy images of mutant *E. coli* strains after biosynthesis conditions. Red arrows show minicells displaying fluorescence emission. Fluorescence images were captured after excitation with a 330–380 nm filter

Further examination of microscopy images reveals that minicells generated in mutant strains exhibit fluorescence emission, indicating their loading with CdS nanoparticles (Fig. 3, red arrows). Given that minicell generation occurs through polar division, this observation suggests that, during the biosynthesis process, nanoparticles situated at (or translocated to) cell poles are encapsulated within forming minicells for subsequent disposal from the cell.

**Fig. 4:**
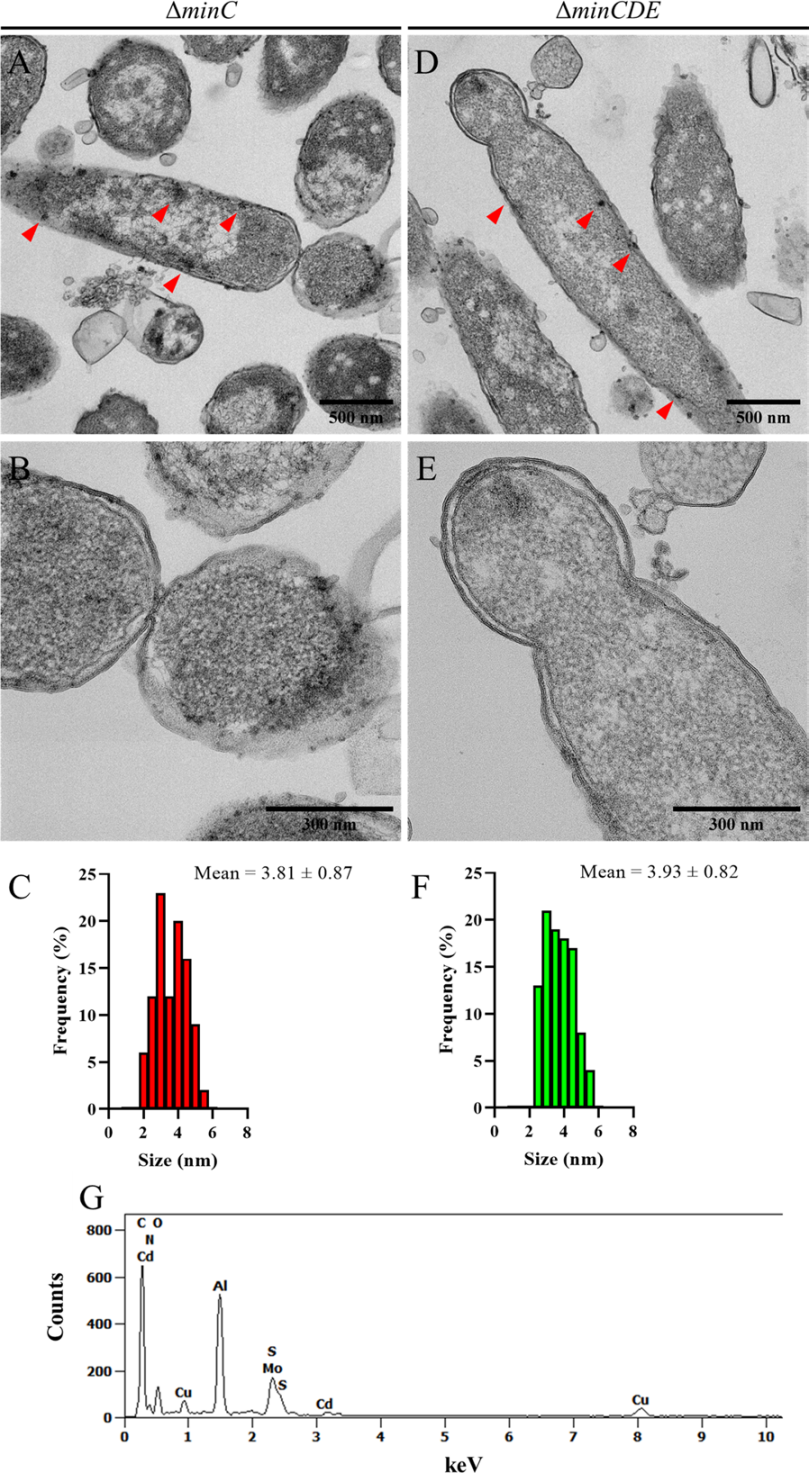
Nanoparticle localization in Min deficient cells. **A**, **D** Representative TEM micrographs of Δ*minC* and Δ*minCDE* strains after biosynthesis conditions. Red arrows show spots with electron-dense nanoparticles inside the cell. **B**, **E** Digital zoom of images (**A**) and (**D**), respectively, highlighting minicells in formation. **C**, **F** Size histograms of nanoparticles inside minicells of (**B**) and (**E**), respectively. **G** EDS analysis of a minicell from Δ*minC* strain after biosynthesis conditions

To confirm the presence of nanoparticles inside minicells and analyze the ultrastructure of cells exposed to CdCl_2_, mutant strains were examined using transmission electron microscopy (TEM). Cells from both strains exhibited electron-dense nanoparticles distributed throughout the cell, predominantly associated with the cell membrane (Fig. 4A, D), consistent with fluorescence microscopy images and fluorescence emission from rod cells (Figs. 2Bc, Bd, 3). Additionally, nanoparticles located at cell poles were found to be encapsulated in minicells (Fig. 4B, E). Similar to rod cells, nanoparticles within minicells are associated with the cell membrane, encompassing both the inner and outer membranes, as well as the periplasmic space. The positioning of nanoparticles inside minicells suggests a sequential process in which nanoparticles are initially synthesized directly at the poles of rod cells and subsequently expelled into minicells. However, electron-dense material from the cytoplasm was also observed, indicating the potential relocation of nanoparticles to cell poles before minicell loading. The size of the electron-dense nanoparticles inside minicells was measured and presented in size histograms (Fig. 4C, F). These nanoparticles exhibited a monodisperse distribution, with diameters ranging between 2 and 6 nm, characteristic of CdS nanoparticles [45]. The presence of cadmium inside minicells was further confirmed through EDS analysis (Fig. 4G), revealing an atomic proportion of 0.15% cadmium inside minicells. Peaks of metals like aluminum or copper can be attributed to the grid used for the analysis [46, 47]. Our findings suggest that, during the biosynthesis of CdS nanoparticles in Min-deficient mutants, a fraction of the nanoparticles becomes associated with the generated minicells and is subsequently expelled from the rod cell.

To complement our analysis, we examined the ultrastructure of strain BW25113 under the same biosynthesis conditions (Additional file 1: Figure S4). Consistent with the findings from fluorescence microscopy analysis (Fig. 2Ba, Bb), the majority of electron-dense material is situated within an extracellular matrix. Intracellularly, some nanoparticles are observable in the periplasmic space (Additional file 1: Figure S4A, indicated by red arrows). Particularly noteworthy is the presence of large electron-dense material at the cell pole, accompanied by a noticeable loss of membrane integrity. A closer examination of this area reveals that the substantial electron-dense material is composed of individual nanoparticles (Additional file 1: Figure S4B). This outcome suggests that, although the cell responds to cadmium presence by synthesizing nanoparticles, the system overwhelms the cell, leading to a loss of integrity. This is in contrast to mutant strains that generate minicells, expelling nanoparticles primarily concentrated at the cell pole.

### CdS nanoparticles are accumulated inside minicells

**Fig. 5:**
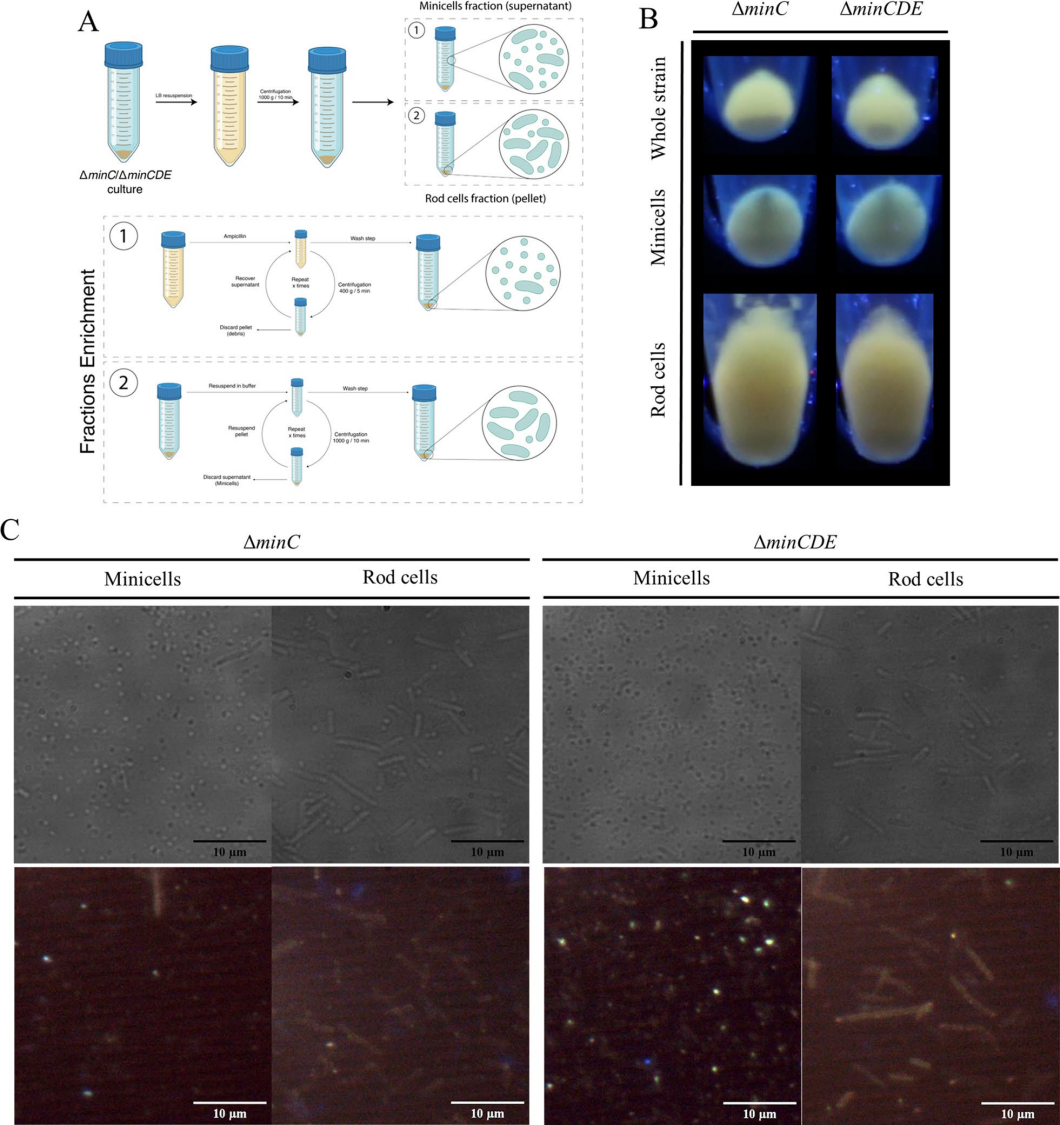
Minicell enrichment. **A** Diagram of the separation process of minicells used in this work. Full description of the process is included in “Materials and Methods”. **B** Cell pellets of mutant strains exposed to UV light (365 nm) for fluorescence detection. Fractions of the whole strain (previous to separation), minicells and rod cells are shown. **C** Representative microscopy images of enriched fractions of minicells and rod cells. Fluorescence microscopy images were captured after excitation with a 330–380 nm filter

As mentioned earlier, the loading of QDs inside minicells could result from the localization of nanoparticles prior to polar cell division. This behavior has been previously characterized as a means of disposing of misfolded proteins from the cell [34]. Considering this, our objective was to explore the capacity of minicells to accumulate fluorescent nanoparticles. To achieve this, we devised a protocol for minicell and rod cell enrichment based on differential centrifugation (Fig. 5A, full description in “Materials and Methods”). After preparing the different fractions following 10 h of biosynthesis, these were pelleted and visualized under UV light. Both minicell and rod cell fractions retained their fluorescence emission after the enrichment process (Fig. 5B), indicating that the procedure did not impact the nanoparticle fluorescence. Microscopy analysis was employed to assess the purity of minicells and rod cells fractions. All fractions were predominantly enriched in their respective cell types, and fluorescence emission was detectable when exposed to UV light, although some minicells exhibited less fluorescence than others (Fig. 5C). This variation may indicate the dynamic nature of the process, as reported in studies suggesting that the biological synthesis of cadmium nanoparticles is not a uniform process [17, 23, 48]. Therefore, it is possible that not all minicells contain the same quantity of nanoparticles.

**Fig. 6:**
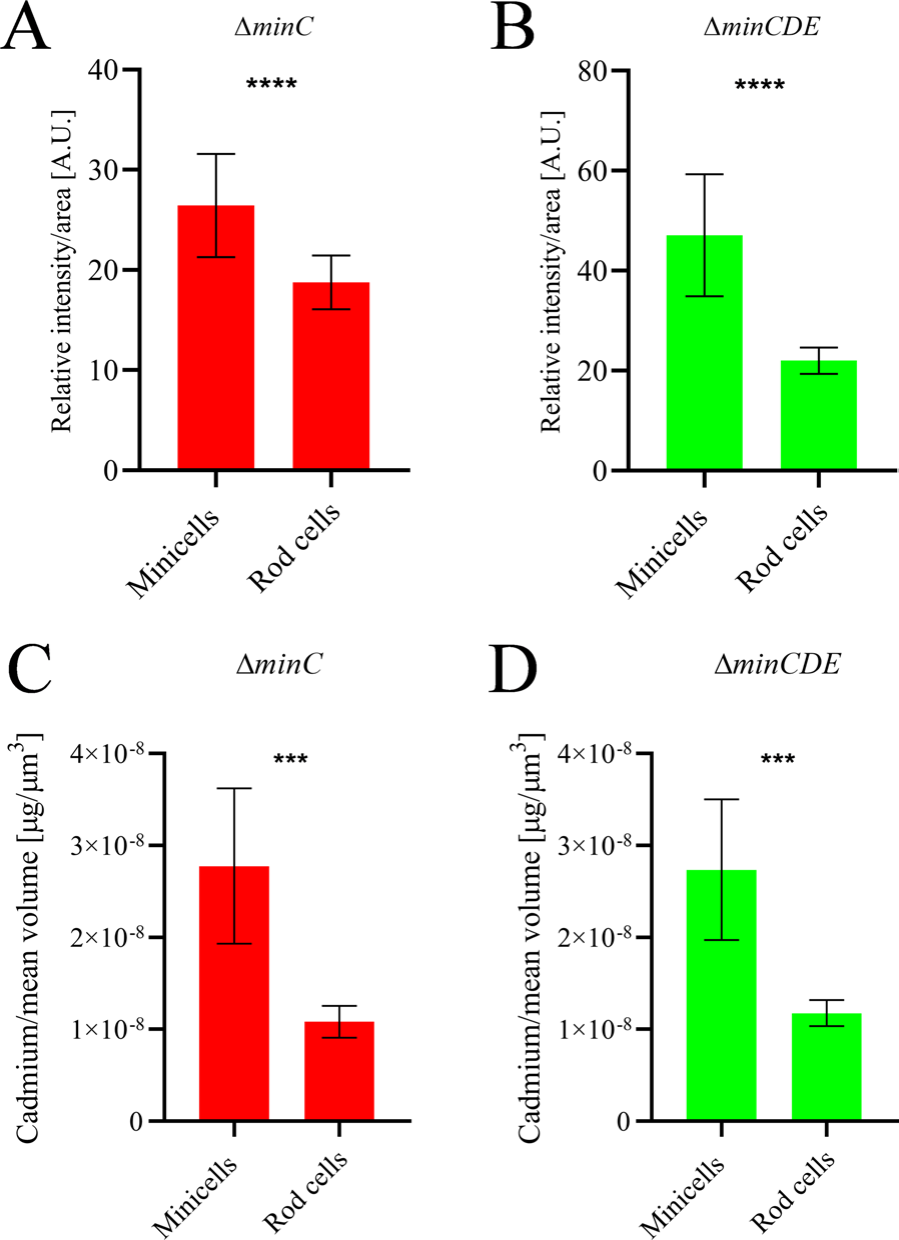
Cadmium quantification inside minicells and rod cells. **A** Quantification of relative fluorescence intensity normalized by area of minicells and rod cells from Δ*minC* strain (n = 30)*.* **B** Quantification of relative fluorescence intensity normalized by area of minicells and rod cells from Δ*minCDE* strain (n = 30)*.* **C** Quantification of cadmium content of minicells and rod cells from Δ*minC* strain, normalized by mean volume. **D** Quantification of cadmium content of minicells and rod cells from Δ*minCDE* strain, normalized by mean volume. Results are expressed from three independent measurements. Statistical analysis was performed by an unpaired two-sided t-test. ***p < 0.001 ****p < 0.0001

Notably, minicells exhibited a higher fluorescence emission compared to rod cells, suggesting the ability of minicells to accumulate nanoparticles. To quantify this, we examined the content of fluorescent nanoparticles in minicells and rod cells by evaluating the difference in pixel intensity, measured from fluorescence microscopy images (Fig. 5C). Results demonstrated that in both strains, minicells displayed a higher relative fluorescence intensity compared to rod cells (Fig. 6A, B). This implies that, during the biosynthesis process, a substantial quantity of actively fluorescent nanoparticles is being loaded inside minicells, potentially serving as a means of nanoparticle disposal from the rod cell. Furthermore, cadmium content in different fractions was assessed by FAAS. Given that minicells have a smaller volume compared to rod cells, we normalized the cadmium content by calculating the mean volume of rod cells and minicells (full description in "Materials and Methods"). The analysis revealed that minicells presented a higher cadmium content per volume (Fig. 6C, D). This not only corroborates the presence of cadmium inside minicells but also suggests that minicells function as a mechanism for eliminating cadmium from the cell.

### Minicell-formation phenotype slows down the biosynthesis process in *E. coli*

**Fig. 7:**
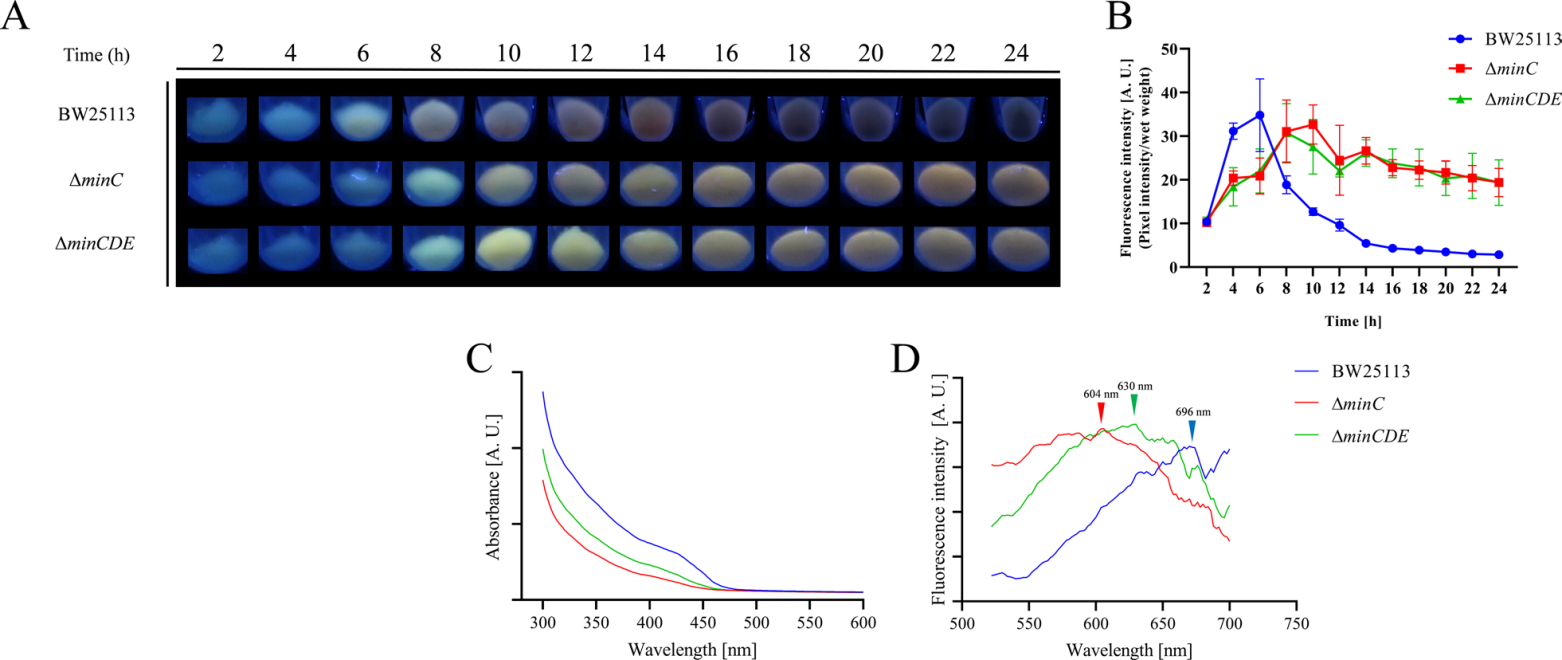
Biosynthesis dynamics in minicell-producing strains. **A** Biosynthesis kinetics of strains BW25113, Δ*minC* and Δ*minCDE.* Strains were grown under biosynthesis conditions at indicated times. Pellets were exposed to UV light (365 nm) for detection of fluorescence. **B** Relative fluorescence intensity of the indicated strains after biosynthesis conditions at different times, analyzed from three independent experiments. **C** Absorbance and **D** fluorescence emission spectra of nanoparticles purified after 10 h of biosynthesis

Our findings demonstrate the loading of fluorescent nanoparticles inside minicells during the biosynthesis process, showcasing a higher accumulation of actively fluorescent nanoparticles compared to rod cells. This variation in biosynthesis suggests potential differences in the kinetics of biosynthesis between minicell-producing strains and the parental *E. coli* strain. To investigate this, we monitored the time-dependent changes in color emission from strains Δ*minC* and Δ*minCDE*, comparing them with the parental BW25113 strain over 24 h. Control reactions without the addition of cadmium showed that cell pellets did not exhibit fluorescence throughout 24 h of growth (Additional file 1: Figure S5), indicating that any changes in fluorescence emission is a result of nanoparticle biosynthesis. Fluorescence emission from BW25113 cells initiates within 4–6 h of the reaction, exhibiting green color emission. This color shifts to yellow and subsequently to orange at 8 and 10 h, respectively. Finally, a transition to red color emission occurs at 14 h, followed by the appearance of black deposits in the pellets (Fig. 7A). These color emission changes align with the classical kinetics of CdS nanoparticle biosynthesis, indicating nanoparticle growth, loss of the quantum confinement effect, and the formation of insoluble cadmium deposits [18, 47, 48]. In contrast, both mutant strains exhibit pellet fluorescence emission beginning after 8 h of biosynthesis. Despite undergoing similar color changes, transitioning from green to yellow and then orange, the orange emission persists from the 14-h mark up to 24 h. The relative fluorescence intensity changes over time were also assessed (Fig. 7B). The relative intensity of strain BW25113 peaks at 6 h, coinciding with the onset of biosynthesis, after which the fluorescence intensity starts to decline. Although the pellet shows fluorescence emission of a defined color, the low fluorescence intensity indicates that only a small amount of nanoparticles remain actively fluorescent, highlighting the rapid growth of the QDs in size. The mutant strains reach maximum intensity at 8–10 h, also correlated with the start of biosynthesis. However, the intensity of mutant strains remains constant up to 24 h, concurrent with the emission color. These findings suggest that the biosynthesis process in minicell-producing strains is comparatively slower than in the parental strain, resulting in a more sustained color change and fluorescence intensity over time. One plausible explanation for this could be that once nanocrystals are loaded inside minicells, their growth ceases, thereby prolonging the overall biosynthesis process. This phenomenon may arise from a missing effector in minicells. One extensively studied enzyme activity involved in bacterial nanoparticle biosynthesis is cysteine desulfhydrase activity, which facilitates the conversion of cysteine-rich proteins to S^−2^ for cadmium sulphide synthesis [49, 50]. To assess if minicells possess cysteine desulfhydrase activity, a sulfur detection assay was performed on enriched fractions of rod cells and minicells. Both cell type fractions exhibited cysteine desulfhydrase activity (Additional file 1: Figure S6B), indicating the presence of another unknown factor possibly associated with the synthesis or growth of nanoparticles that might be absent in minicells. While some residual rod cells are identified in the minicell purification process (Additional file 1: Figure S6A), their short size in comparison to the purified rod cells and the antibiotic treatment in the process (see "Materials and Methods") suggest that these may be potentially non-viable cell fragments and debris [39]. Importantly, these elements should not contribute to cysteine desulfhydrase activity in the minicell fraction.

To assess whether nanoparticles synthesized in the same incubation time exhibit optical properties representative of different sizes, nanoparticles were purified from pellets of the three strains after 10 h of biosynthesis, and absorption and fluorescence emission spectra were analyzed. While all absorption spectra display a characteristic peak above 400 nm (Fig. 7C), indicative of nanoparticles with similar characteristics, fluorescence emission peaks suggest a difference in size among nanoparticles from each strain. Strain BW25113 exhibits a peak above 650 nm, whereas mutant strains display peaks between 550 and 650 nm, indicative of a smaller size.

## Discussion

Some cellular functions enable bacteria to tolerate certain concentrations of heavy metals. At first, bacteria can sequester metal ions adsorbed into their extracellular substances [51, 52]. Once metals are inside the cell, they can be expelled by efflux systems. For instance, *Enterococcus hirae* possesses CopA and CopB ATPases capable of transporting monovalent metal cations (copper and silver) out of the bacterium [53]. In *E. coli* the ATP-dependent transporter ZntA releases zinc and cadmium to the periplasmic space for subsequent excretion [20, 54, 55]. Additionally, CadA functions as an efflux ATPase, regulating the intracellular levels of cadmium and other metals, and is regulated by the cadmium-binding proteins CadR in *Pseudomonas putida* [56] and CadC in *Staphylococcus aureus* [57]. Sequestration of heavy metals inside the cell is another strategy involving adsorption by cell-produced molecules like metallothioneins, peptides rich in cysteine capable of trapping metal ions [58–60]. The biosynthesis of nanoparticles represents a novel defense mechanism against heavy metals. Metal accumulation leads to their mineralization into various structures, typically small particles. Bacterial exposure to heavy metals has been observed to result in the formation of electron-dense structures both inside [61] and outside [62] the cell, as well as structures associated with bacterial membranes [63]. However, understanding how cell structures influence these processes to enhance the cell's response to heavy metals remains limited.

Recent research has explored the role of minicells in bacterial stress tolerance. Rang et al. [34] demonstrated that minicell formation in *E. coli* enhances tolerance to misfolded protein stress by sequestering inclusion bodies inside minicells. Furthermore, Kim & Oh [64] demonstrated that the minicell-producing phenotype has higher tolerance to different toxic chemicals for *E. coli*, such as isobutyraldehyde, isobutanol or isobutyl acetate. Although the relocation of damage elements in *E. coli* has been studied and a model for cell aging has been proposed [65], the molecular mechanisms underlying this process and whether it is triggered by different stress types remains unknown. In light of these findings, we aimed to investigate whether minicells could serve as a mechanism for nanoparticle elimination. To address this, we examined whether nanoparticle biosynthesis in minicell-producing strains enhances metal disposal through polar localization and encapsulation in minicells.

Using deletion mutants of *E. coli* Δ*minC* and Δ*minCDE*, known models for minicell production (Fig. 1), we implemented a standardized biosynthesis protocol [19]. Since minicell production is directly associated with cell division, the biosynthesis of QDs during cell growth is ideal for our study. As seen in Fig. 2A, the exposure of cells to CdCl_2_ during growth induces the biosynthesis of QDs in both mutant strains, as well in the parental BW25113 strain. As expected, the morphological analysis of BW25113 strain showed cell aggregation and high fluorescence emission at points of cell interaction (Fig. 2Ba, Bb). This behavior was previously analyzed by our group, and we deduced that the biosynthesis process is related to the formation of an extracellular matrix for the elimination of the metal from the cell (unpublished results). In contrast, Δ*minC* and Δ*minCDE* cells displayed fluorescence emission primarily from individual cells (Fig. 2Bc, Bd), with only a small fraction of cells forming cell aggregates (Additional file 1: Figure S1). Furthermore, we detected that nanoparticles are being loaded inside minicells (Fig. 3). This result suggests that nanoparticles are loaded into minicells during cell division and, although the biosynthesis process should be occurring through the same mechanisms as in the parental strain, the minicell formation phenotype has an additional effect on the way the cell responds to the presence of cadmium. This is consistent with the damage-disposal role of minicells, as analyzed by the elimination of damaged proteins [34]. The ultrastructure of Min mutants after biosynthesis conditions revealed that nanoparticles inside minicells are associated primarily with the cell membrane (Fig. 4). These nanoparticles can be observed both in the periplasmic space and the inner membrane (Fig. 4B, E). This could suggest that a fraction of the nanoparticles is being synthesized in the membrane of the cell pole for their subsequent elimination in minicells, while another fraction is being synthesized in the cytoplasm and then translocated to the cell pole.

As we previously mentioned, minicells have been studied for their ability to eliminate damaged proteins from the cell [34]. Given that our results indicate the loading of cadmium nanoparticles inside minicells as well, we sought to investigate the ability of minicells to dispose of cadmium through this mechanism. To accomplish this, we employed an enrichment method that yielded highly enriched fractions of minicells and rod cells (Fig. 5). Quantification of relative fluorescence and total cadmium by FAAS revealed that minicells from strains Δ*minC* and Δ*minCDE* accumulate a higher amount of fluorescent nanoparticles than rod cells (Fig. 6), suggesting that minicells generated in Min mutant strains function as a mechanism for the disposal of cadmium from the cell. Previously, it was studied how inclusion bodies formed by streptomycin exposure in *E. coli* were relocalized to the cell poles, probably due to interaction with chaperone proteins [34]. A similar phenomenon could be occurring with intracellular cadmium nanoparticles, which present a coating of organic matter, including proteins and other biomolecules when synthesized using bacteria [25]. The primary damaging effect of cadmium exposure in *E. coli* is the formation of misfolded proteins due to binding to thiol groups and disruption of iron-sulfur clusters, inducing the up-regulation of genes involved in protein refolding or degradation [9, 16, 35].This allows us to hypothesize that biosynthesized nanoparticles are coated with damaged proteins, which could be relocalized to cell poles by chaperones or other unknown factors. Several reports have studied the preferential localization of proteins using minicell-producing strains of *E. coli*. Through proteomic approaches, different proteins have been found enriched at cell poles, such as porin-like outer membrane proteins, chaperones and several other enzymes [38, 66]. Furthermore, studies suggest that several cell proteins can directly interact with the components of the Min system, either through recruitment or exclusion [67]. This implies that Min system oscillations (or the lack of them) can affect the distribution of proteins in the cell. Additionally, other factors have been studied regarding the localization of molecules inside the cell. The post-transcriptional localization of RNAs has been investigated in *E. coli*, revealing that several stress-related mRNAs and small RNAs are preferentially located in the pole of the cell [68], supporting the notion of cell poles as hotspots for damage accumulation. Moreover, new protein factors have been studied for their specific capacity of protein relocalization to cell poles [69]. Further studies in our group are underway to elucidate the molecular factors involved in the intracellular distribution of nanoparticles and their subsequent disposal inside minicells.

As previously analyzed, morphological differences between parental strain BW25113 and Min mutants suggest that the minicell-forming phenotype could be influencing the cell’s response to the biosynthesis conditions (Fig. 2). In this biosynthesis system, the sulfate present in the M9 medium serves as the sulfur source that is incorporated into the cell and subsequently metabolized to produce H_2_S [70]. Due to the high affinity of cadmium for sulfur [9], fluorescent CdS nanoparticles are formed. With increasing incubation time, larger nanoparticles develop as more cadmium ions are incorporated into the nanostructure [19]. However, the quantum confinement effect comes into play; when nanoparticles become too large, the electronic energy levels cease to be discrete, leading to the cessation of fluorescence emission [6]. As a consequence, many bacterial biosynthesis systems follow kinetics resulting in the formation of black cadmium deposits during prolonged incubation times. When we examined the differences in biosynthesis kinetics among our strains, we observed that strain BW25113 adheres to the expected kinetics of quantum dots biosynthesis (Fig. 7A). Morphological analysis of this strain following quantum dots biosynthesis (Additional file 1: Figure S4) reveals tightly joined individual crystals, forming large, electron-dense structures. Notably, synthesis in the Min mutant strains exhibits important differences: Biosynthesis commences after a longer incubation time, and fluorescence intensity persists even after 24 h of incubation (Fig. 7A, B). This suggests that nanoparticle synthesis is slower in mutant strains, requiring an extended incubation time for the evolution of fluorescence color. This is corroborated by disparities in fluorescence emission spectra (Fig. 7D), indicating that nanocrystals synthesized in strain BW25113 grow faster than those in mutant strains within a defined assay time. Although it is reported that Min mutants have slower growth rate than wild type strains due to the formation of minicells [34], there is no evidence that these strains are affected in other metabolic functions that could be involved in the synthesis of QDs. In fact, our results suggest that minicells possess cysteine desulfhydrase activity (Additional file 1: Figure S6), an enzymatic activity extensively studied due to its involvement in bacterial nanoparticle biosynthesis [19, 49, 50]. Therefore, it is possible that when crystals are loaded inside minicells, they cease growing due to the absence of an, as yet unknown, factor involved in nanoparticle biosynthesis, resulting in the observed slower overall fluorescence emission. This is consistent with the comparison of relative fluorescence between minicells and rod cells, where minicells exhibit higher relative intensity (Fig. 6A, B). This difference may be attributed not only to the higher cadmium content (Fig. 6C, D) but also to the possibility that nanoparticles inside rod cells are larger, displaying lower fluorescence intensity. This behavior, in comparison to what occurs in the BW25113 strain, suggests that minicell disposal of nanoparticles works as an additional defense mechanism against cadmium exposure and avoids an “overrun” by eliminating immobilized cadmium ions.

Upon exposing *E. coli* cells to both biological and chemical CdS nanoparticles and monitoring their growth, we observe that although biological nanoparticles are less toxic than chemical nanoparticles, cell growth is impaired with increasing concentrations of QDs (Additional file 1: Figure S7). It's also important to note that the described mechanism for metal nanoparticle toxicity in cells requires contact with the cell membrane [11, 12]. This suggests that minicell-mediated nanoparticle removal not only aids the cell by eliminating the nanoparticles but also by encapsulating them in a lipid bilayer, thus preventing direct contact with viable rod cells.

Our results prompt the question of how the formation of minicells could aid in the detoxification of cadmium in the form of nanoparticles from wild type cells, particularly in the absence of mutations that promote minicell formation. It is well known that minicell formation in wild type cells is exceedingly rare and challenging to detect [71], underscoring the difficulty in studying this phenomenon in cells lacking mutations in the Min system. However, morphological analyses conducted in various QD biosynthesis systems suggest the possibility that minicell production is linked to the synthesis of cadmium nanoparticles. Several studies have indicated that intracellular nanoparticles are primarily associated with the cell membrane and poles in various QD biosynthesis systems. In a strain expressing a CdS binding peptide, nanostructures are associated with the cell membrane and cell poles as evidenced by TEM [66]. A similar behavior was observed in the work by Marusak et al. [48] where the CdS nanoparticles synthesized by a strain expressing an heterologous cysteine desulfhydrase gene were located primarily associated with cell membranes. In an *E. coli* system for the biosynthesis of cadmium and selenium QDs, the presence of nanoparticles was mainly located in the cell poles [20]. Interestingly, this phenotype also occurs on other gram-negative bacteria, like species of the *Pseudomonas* and *Psychrobacter* genera. When synthesizing CdS QDs in these species, nanomaterials are accumulated near the cell poles and morphological changes are detectable in said poles, like a loss of integrity of cell membrane [67] or widening of the periplasmic space [44], which supports the proposal of a cell division process involved with the biosynthesis of QDs. Our previous work demonstrated that under biosynthesis conditions in *E. coli*, a minicell-like phenotype is detectable, suggesting a connection between minicell formation and nanoparticle biosynthesis [23]. The molecular mechanism underlying minicell formation could potentially be mediated by a general down-regulation of genes involved in cell division as a consequence of cadmium exposure [72]. Exposure of *E. coli* cells to cadmium ions and CdS nanoparticles has been shown to induce cell filamentation and inhibit the correct formation of the division septum, which involves a decrease in the expression of FtsZ and FtsQ division proteins [73, 74]. These proteins play a crucial role in the formation of the “Z-ring” at the center of the cell [75]. In summary, these antecedents suggest that cadmium exposure and/or biosynthesis of cadmium nanoparticles can affect cell division as a means for minicell formation and subsequent cadmium elimination from the cell.

**Fig. 8:**
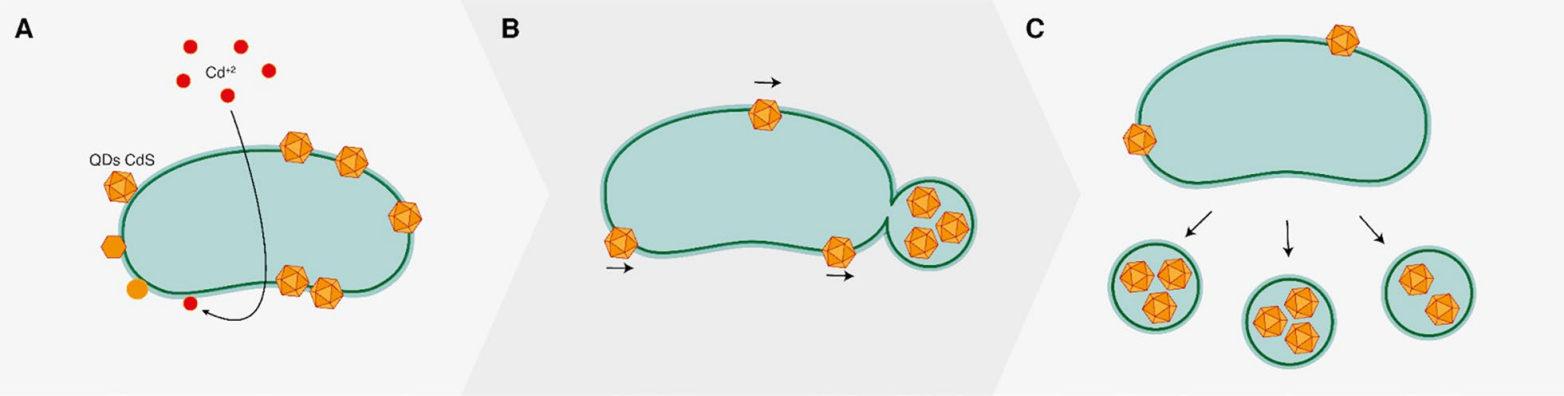
Proposed model for the accumulation and elimination of nanoparticles through minicells. **A** *E. coli* cells synthesize nanoparticles as means for metal immobilization and tolerance. These nanoparticles are associated to the cell membrane and are distributed through the cell. **B** A fraction of nanoparticles is relocated to a cell pole. Minicell formation causes the encapsulation of nanoparticles, eliminating them from the cell. **C** Continuous elimination of nanoparticles from the cell inside minicells promotes cell fitness and allows other metal tolerance mechanisms to be bypassed

Taken together, our results enable us to propose a model of the disposal of fluorescent cadmium nanoparticles inside minicells (Fig. 8). Cadmium nanoparticles appear to be synthesized in response to cadmium ions, a process previously described in detail [19]. This synthesis involves the uptake of metal ions through phosphate transporters and sulfur metabolism. A portion of the nanoparticles relocates to the cell poles. Subsequently, these nanoparticles and membrane-bound counterparts become encapsulated in developing minicells and are subsequently expelled from the entire cell. Due to the constitutive nature of minicell formation in Min mutants, the elimination of nanoparticles becomes a continuous process, potentially bypassing other anticipated cellular responses to the metal, such as biofilm formation or the exopolysaccharide trapping of nanoparticles [76]. In this context, our study represents the first report unveiling the physiological role of minicells as a mechanism for heavy metal tolerance.

Regarding the biotechnological significance of our findings, our results suggest that minicell formation in bacteria enables better control of the optical properties of the resulting nanoparticles (Fig. 7). This is noteworthy, especially considering that a multitude of bacterial species, isolated from various sources, have recently been utilized for the biosynthesis of high-quality QDs with distinct properties and under simple reaction conditions [44, 77–79]. Considering this, having access to an improved method that allows for size control of nanoparticles is a crucial avenue for further exploration. Furthermore, minicell formation has been achieved in both Gram-negative and Gram-positive bacteria [80], indicating that this approach is not limited to *E. coli*.

Another intriguing aspect of our work is the ability to separate and prepare an enriched fraction of nanoparticle-loaded minicells (Fig. 5). One of the most notable challenges with QDs lies in their low biocompatibility and poor water solubility [81]. QDs based on heavy metals such as cadmium or tellurium are highly toxic, and despite progress in synthesis and applications in cell lines [24, 25, 82], they are not yet approved for medical use. This issue also extends to other clinically important nanoparticles, such as upconversion nanoparticles, proposed for novel biomedicine applications but hindered by their chemical characteristics and poor biocompatibility [83]. Our work opens the possibility of obtaining multiple types of nanoparticles through bacterial biosynthesis, producing high-quality nanoparticles encapsulated in minicells to enhance their biocompatibility and potential applications in biomedicine and imaging.

## Conclusions

Our study sheds light on the intricate interplay between bacterial cellular processes and the biosynthesis of cadmium nanoparticles, with a particular emphasis on the role of minicells in heavy metal tolerance. We have demonstrated that minicells, formed in Min mutant strains of *E. coli*, play a crucial role in the disposal of fluorescent cadmium nanoparticles, providing a continuous and efficient mechanism for metal elimination. The encapsulation of nanoparticles within minicells at cell poles reveals a unique strategy employed by bacteria to enhance their response to heavy metal stress. Moreover, our findings present a novel perspective on the biotechnological implications of minicell formation in bacteria. The ability to control the optical properties of synthesized nanoparticles, coupled with the feasibility of isolating enriched fractions of nanoparticle-loaded minicells, opens avenues for improved methods in nanoparticle synthesis with potential applications in biomedicine and imaging. The broader applicability of minicell formation across Gram-negative and Gram-positive bacteria further emphasizes the versatility of this approach beyond *E. coli*. Overall, our study not only advances our understanding of bacterial responses to heavy metal stress but also contributes valuable insights into the potential biotechnological applications of minicells in nanoparticle synthesis, paving the way for further exploration in the realm of nanotechnology and biomedicine.

### Supplementary Information

**Additional file 1: Figure S1.** CdS NPs biosynthesis at different cadmium concentrations. Bacterial pellets exposed to UV light (365 nm) of indicated *E. coli* strains grown on M9-glucose medium supplemented with 10 μg/ml CdCl_2_, 60 μg/ml CdCl_2_ or without metal (control). **Figure S2.** Fluorescence emission in cell aggregates of Min mutants. Representative panoramic images of cells from strains Δ*minC* and Δ*minCDE* after biosynthesis conditions after 14 h at 37 °C. Red arrows highlight cell aggregates. **Figure S3.** Fluorescence microscopy of *E. coli* cells not exposed to cadmium. Representative images from strains BW25113, Δ*minC* and Δ*minCDE* grown in M9-glucose medium in the absence of CdCl_2_ after 14 h at 37 °C. Fluorescence images were captured after excitation with a 330–380 nm filter. **Figure S4.** Nanoparticle localization in *E. coli* BW25113. (A) Representative TEM micrographs of *E. coli* BW25113 wt strain during nanoparticle biosynthesis. Red arrows show spots with electron-dense nanoparticles inside the cell. (B) Digital zoom of (A) showing electron-dense material in the pole of the cell. **Figure S5.** *E. coli* strains exposed to biosynthesis conditions in absence of cadmium. Biosynthesis kinetics of strains BW25113, Δ*minC* and Δ*minCDE* without the addition of CdCl_2_*.* Strains were grown in M9-glucose medium in the absence of CdCl_2_ at 37 °C after the indicated times. Pellets were exposed to UV light (365 nm) for detection of fluorescence. **Figure S6.** Sulphide production from minicells. (A) Optical microscopy images of enriched fractions of minicells and rod cells from Min mutants. (B) Detection of cysteine desulfhydrase activity from minicells and rod cells fractions, evaluated by the apparition of black precipitates on filter paper. A representative assay of three independent experiments is shown. **Figure S7.** Toxicity of different CdS nanoparticles on *E. coli*. Absorbance (blue line) and fluorescence emission (red line) spectra of biological (A) and chemical (B) CdS nanoparticles. Insets of the graphs show solutions of the respective nanoparticles, exposed to UV light (365 nm). (C) Growth curve of *E. coli* supplemented with biological nanoparticles. (D) Growth curve of *E. coli* supplemented with chemical nanoparticles. The concentration of nanoparticles used is indicated in the graph.

## Data Availability

The datasets used and/or analyzed during the current study are available from the corresponding author on reasonable request.

## Abbreviations

LB: Lysogeny broth
CdS: Cadmium sulphide
TEM: Transmission electron microscopy
EDS: Energy-dispersive X-ray spectroscopy
QDs: Quantum dots
ROS: Reactive oxygen species
FAAS: Flame atomic absorption spectrometry
GSH: Glutathione
NA: Numerical aperture
EPS: Extracellular polymeric substances
