# Supplementary material for: Minicells as an Escherichia coli mechanism for the accumulation and disposal of fluorescent cadmium sulphide nanoparticles
Source: J Nanobiotechnology. 2024 Feb 27;22:78. doi: 10.1186/s12951-024-02348-0 (PMC10900627; doi:10.1186/s12951-024-02348-0)
Supplement: Supplementary file 1 — Additional file 1: Figure S1. CdS NPs biosynthesis at different cadmium concentrations. Bacterial pellets exposed to UV light (365 nm) of indicated E. coli strains grown on M9-glucose medium supplemented with 10 μg/ml CdCl2, 60 μg/ml CdCl2 or without metal (control). Figure S2. Fluorescence emission in cell aggregates of Min mutants. Representative panoramic images of cells from strains ΔminC and ΔminCDE after biosynthesis conditions after 14 h at 37 °C. Red arrows highlight cell aggregates. Figure S3. Fluorescence microscopy of E. coli cells not exposed to cadmium. Representative images from strains BW25113, ΔminC and ΔminCDE grown in M9-glucose medium in the absence of CdCl2 after 14 h at 37 °C. Fluorescence images were captured after excitation with a 330–380 nm filter. Figure S4. Nanoparticle localization in E. coli BW25113. (A) Representative TEM micrographs of E. coli BW25113 wt strain during nanoparticle biosynthesis. Red arrows show spots with electron-dense nanoparticles inside the cell. (B) Digital zoom of (A) showing electron-dense material in the pole of the cell. Figure S5. E. coli strains exposed to biosynthesis conditions in absence of cadmium. Biosynthesis kinetics of strains BW25113, ΔminC and ΔminCDE without the addition of CdCl2. Strains were grown in M9-glucose medium in the absence of CdCl2 at 37 °C after the indicated times. Pellets were exposed to UV light (365 nm) for detection of fluorescence. Figure S6. Sulphide production from minicells. (A) Optical microscopy images of enriched fractions of minicells and rod cells from Min mutants. (B) Detection of cysteine desulfhydrase activity from minicells and rod cells fractions, evaluated by the apparition of black precipitates on filter paper. A representative assay of three independent experiments is shown. Figure S7. Toxicity of different CdS nanoparticles on E. coli. Absorbance (blue line) and fluorescence emission (red line) spectra of biological (A) and chemical (B) CdS nanoparticles. Insets [file 12951_2024_2348_MOESM1_ESM.docx]

**Additional file for**

**Minicells as an *Escherichia coli* mechanism for the accumulation and disposal of fluorescent cadmium sulphide nanoparticles**

Felipe Valenzuela-Ibaceta^1^, Nicolás Torres-Olea^1^, Javiera Ramos-Zúñiga^1^, Claudio Dietz-Vargas^1^, Claudio A. Navarro^1^, José M. Pérez-Donoso^1^*.

^1^Universidad Andrés Bello, BioNanotechnology and Microbiology Laboratory, Center for Bioinformatics and Integrative Biology (CBIB), Facultad de Ciencias de la Vida, Av. República # 330, Santiago, Chile.

* Corresponding author: jose.perez@unab.cl


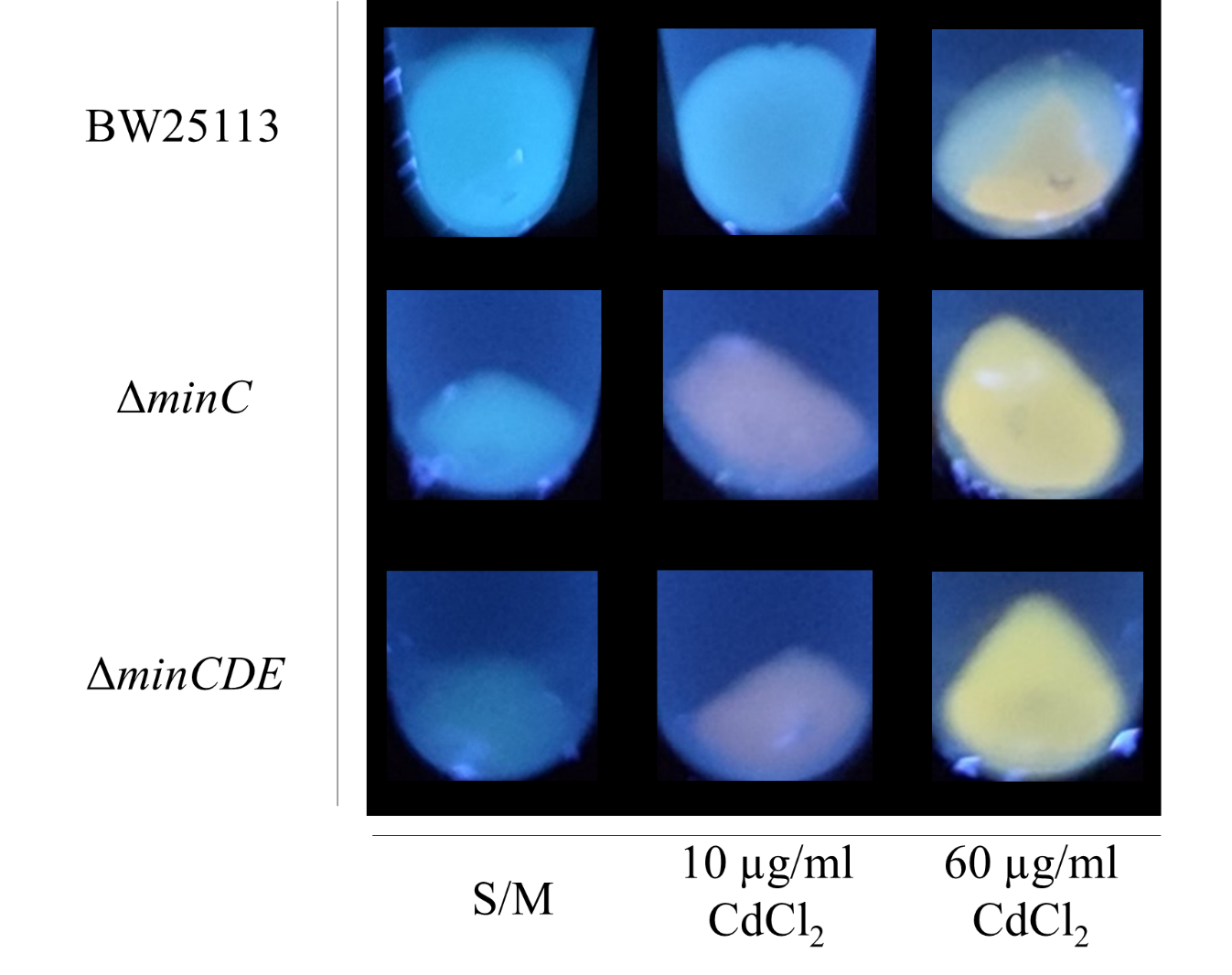


**Figure S1: CdS NPs biosynthesis at different cadmium concentrations.** Bacterial pellets exposed to UV light (365 nm) of indicated *E. coli* strains grown on M9-glucose medium supplemented with 10 μg/ml CdCl_2_, 60 μg/ml CdCl_2_ or without metal (control).


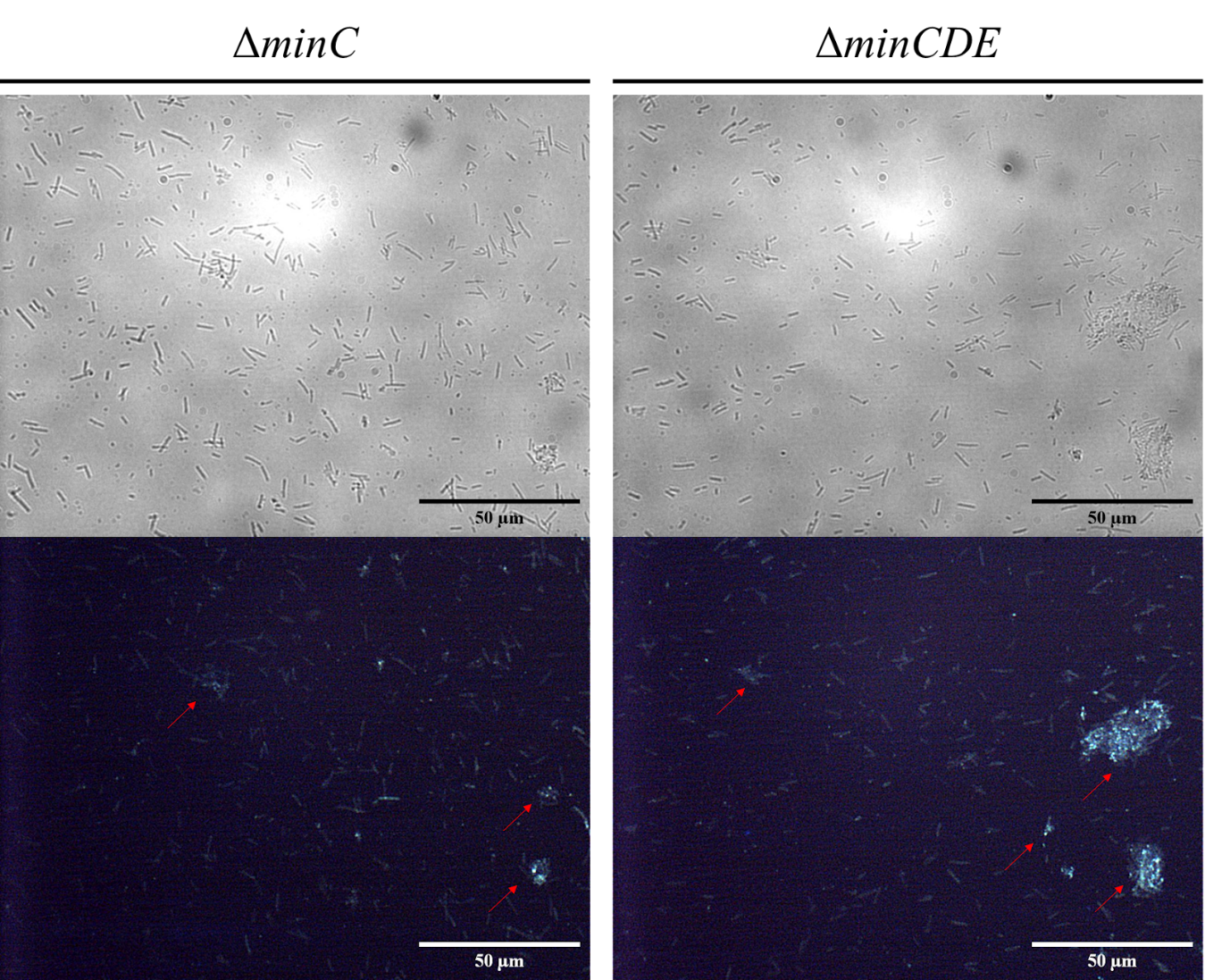


**Figure S2: Fluorescence emission in cell aggregates of Min mutants**. Representative panoramic images of cells from strains Δ*minC* and Δ*minCDE* after biosynthesis conditions after 14 hours at 37°C. Red arrows highlight cell aggregates.


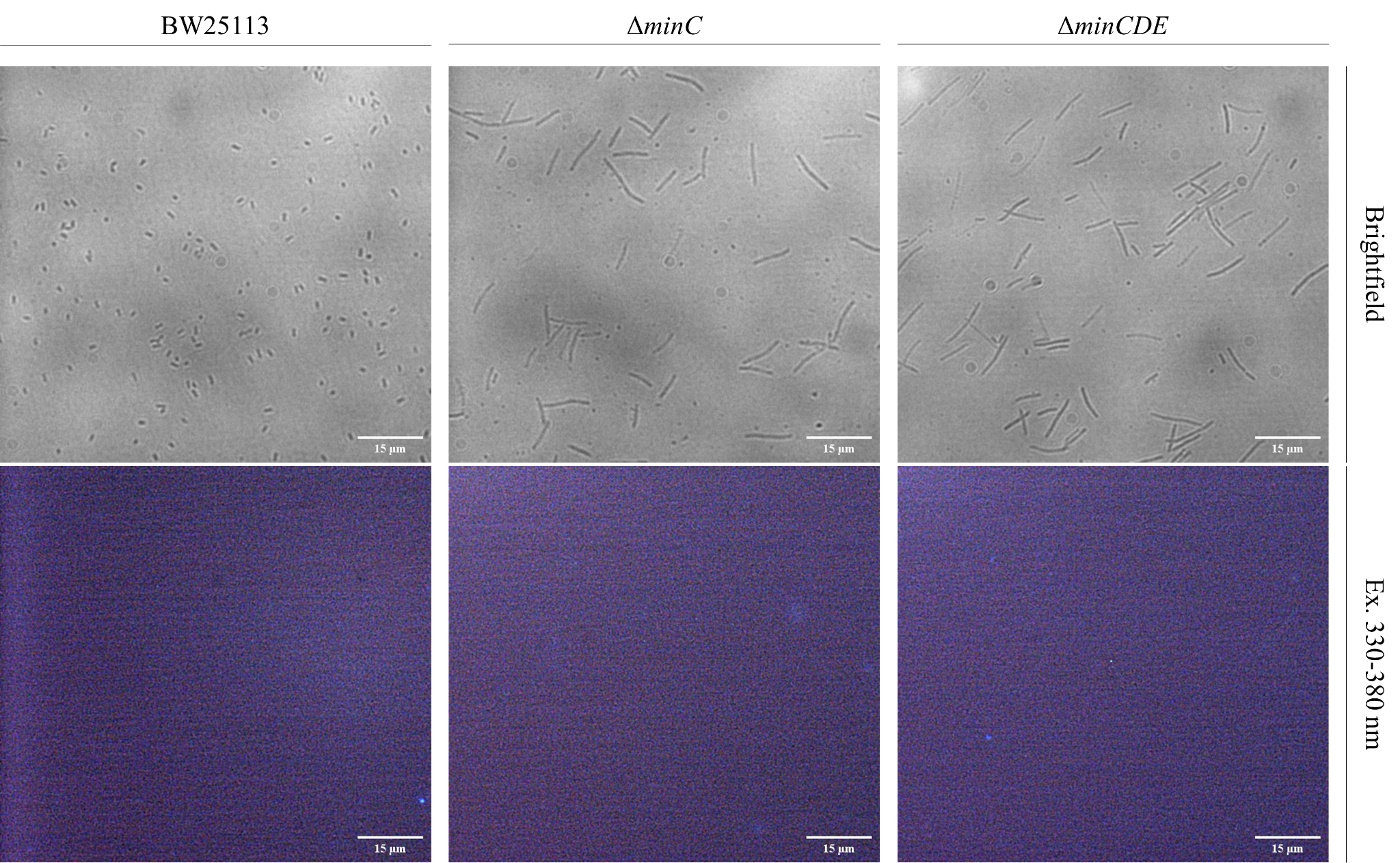


**Figure S3: Fluorescence microscopy of *E. coli* cells not exposed to cadmium**. Representative images from strains BW25113, Δ*minC* and Δ*minCDE* grown in M9-glucose medium in the absence of CdCl_2_ after 14 hours at 37°C. Fluorescence images were captured after excitation with a 330-380 nm filter.


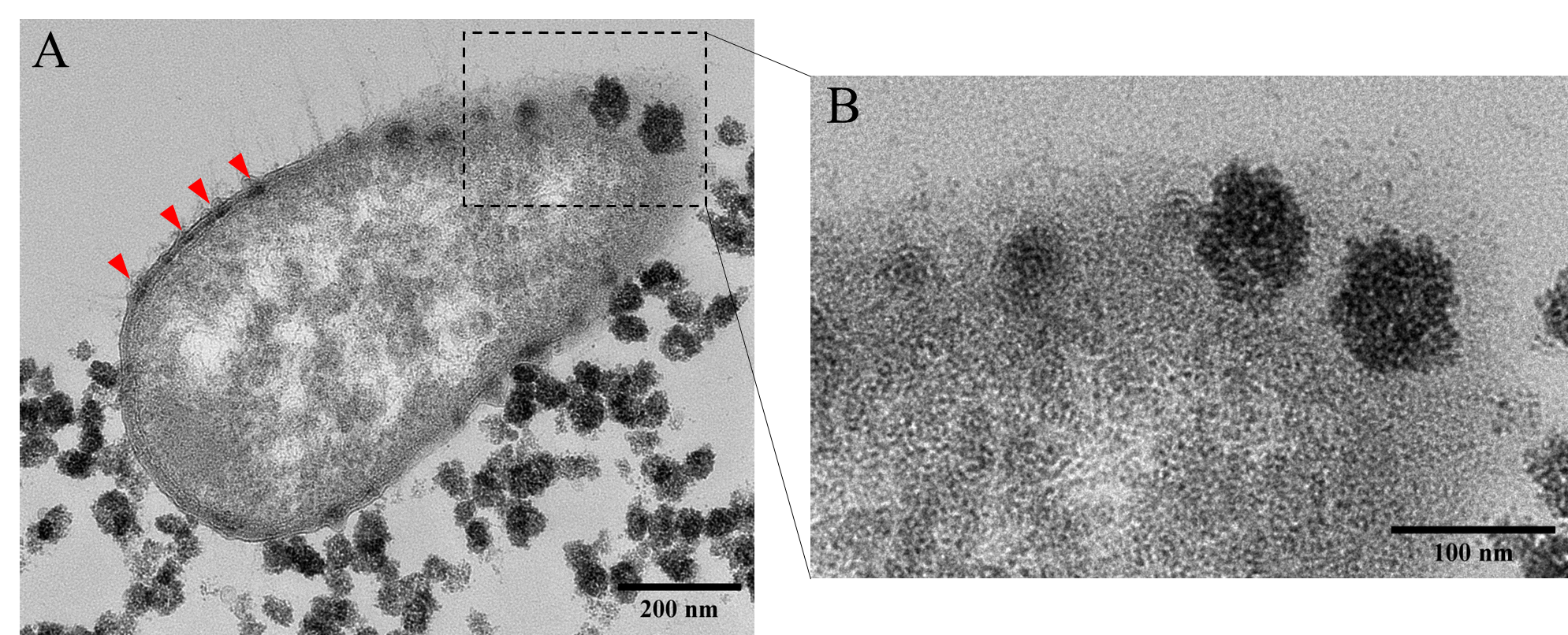


**Figure S4: Nanoparticle localization in *E. coli* BW25113**. (A) Representative TEM micrographs of *E. coli* BW25113 wt strain during nanoparticle biosynthesis. Red arrows show spots with electron-dense nanoparticles inside the cell. (B) Digital zoom of (A) showing electron-dense material in the pole of the cell.


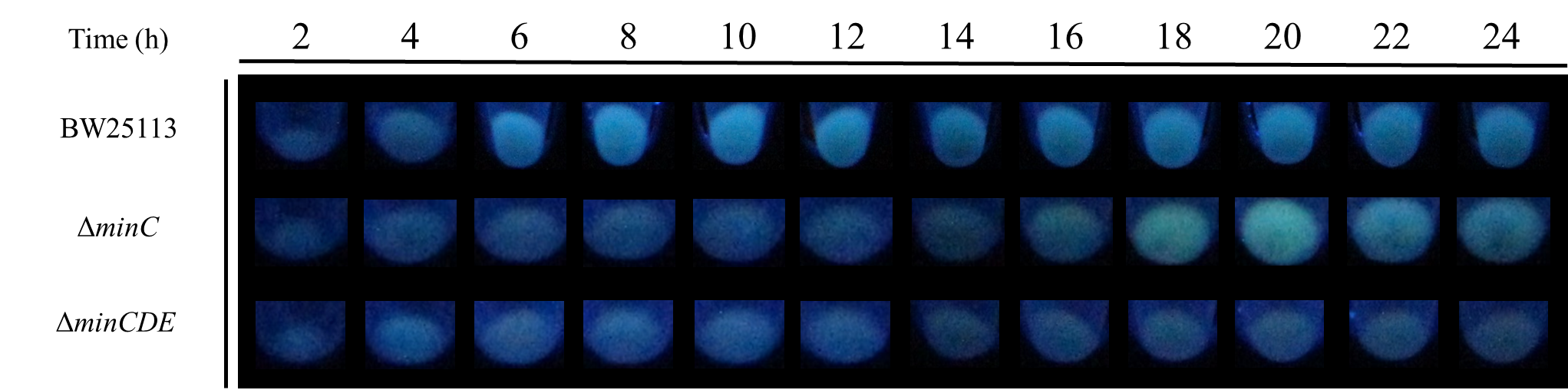


**Figure S5: *E. coli* strains exposed to biosynthesis conditions in absence of cadmium.** Biosynthesis kinetics of strains BW25113, Δ*minC* and Δ*minCDE* without the addition of CdCl_2_*.* Strains were grown in M9-glucose medium in the absence of CdCl_2_ at 37°C after the indicated times. Pellets were exposed to UV light (365 nm) for detection of fluorescence.


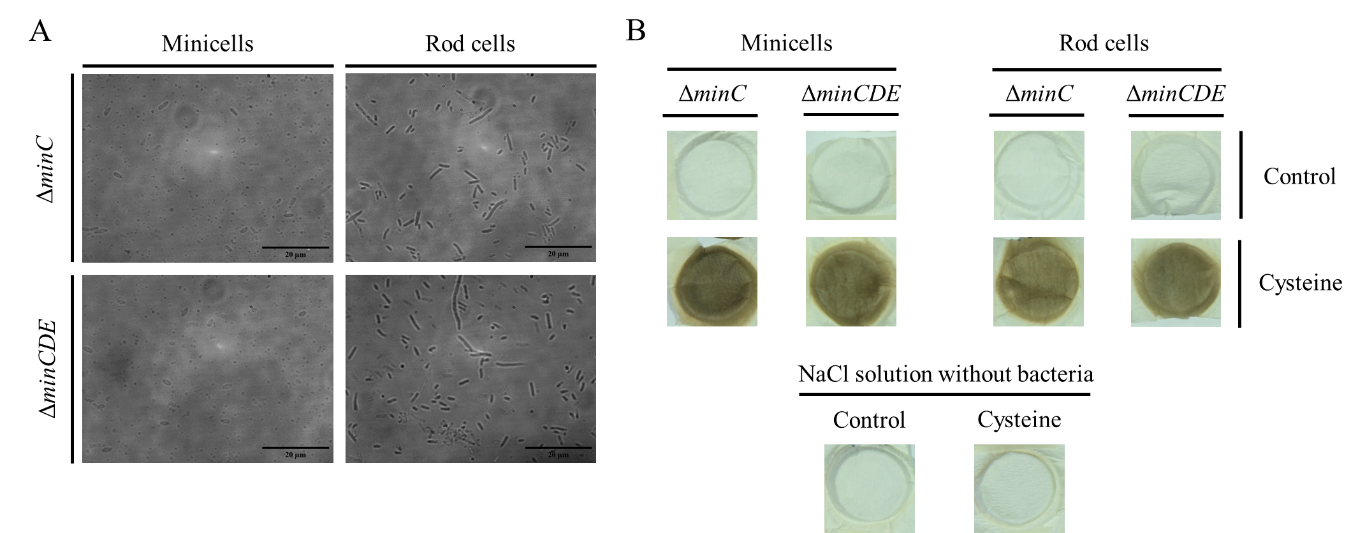


**Figure S6: Sulphide production from minicells**. (A) Optical microscopy images of enriched fractions of minicells and rod cells from Min mutants. (B) Detection of cysteine desulfhydrase activity from minicells and rod cells fractions, evaluated by the apparition of black precipitates on filter paper. A representative assay of three independent experiments is shown.


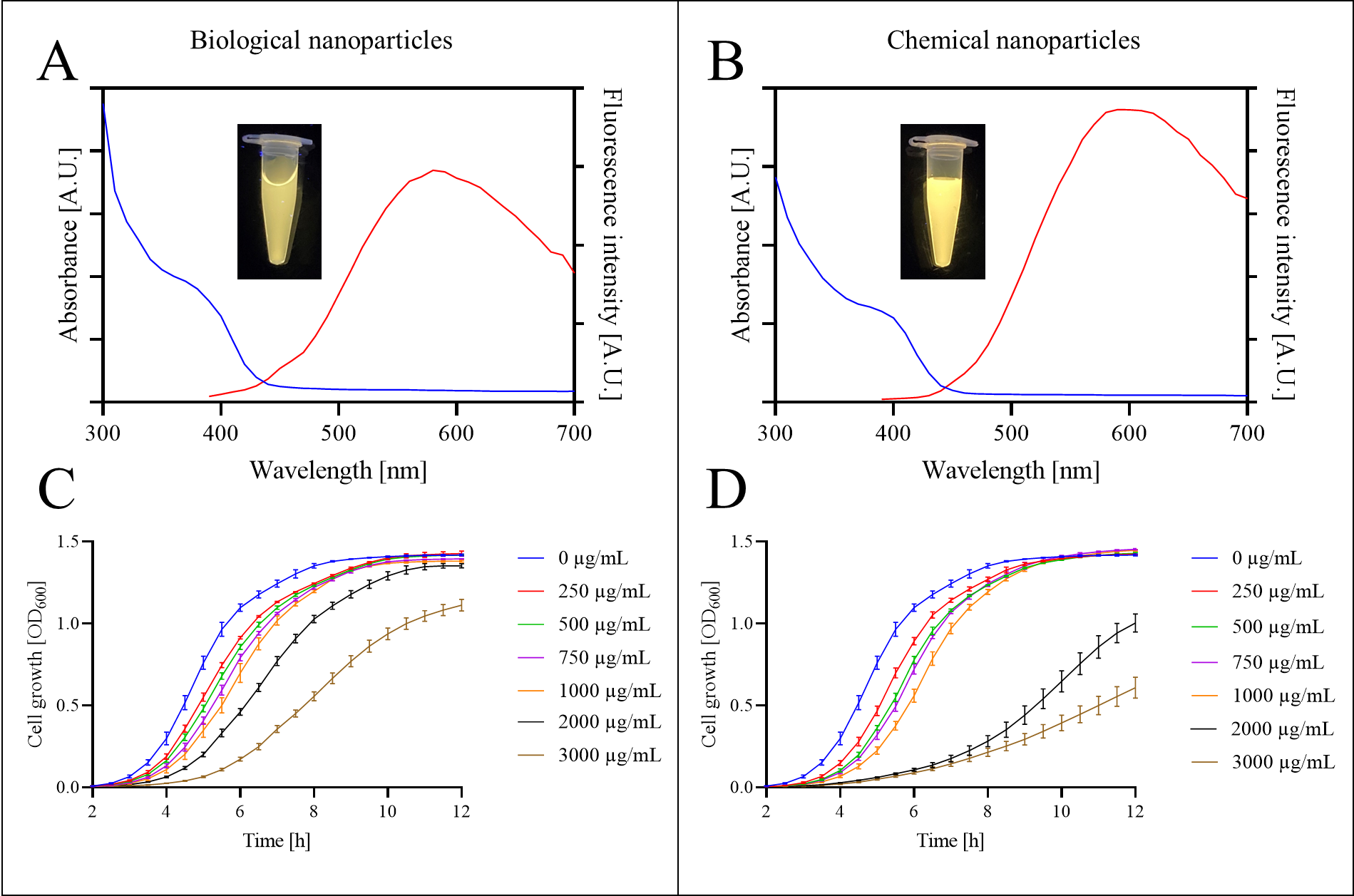


**Figure S7: Toxicity of different CdS nanoparticles on *E. coli***. Absorbance (blue line) and fluorescence emission (red line) spectra of biological (A) and chemical (B) CdS nanoparticles. Insets of the graphs show solutions of the respective nanoparticles, exposed to UV light (365 nm). (C) Growth curve of *E. coli* supplemented with biological nanoparticles. (D) Growth curve of *E. coli* supplemented with chemical nanoparticles. The concentration of nanoparticles used is indicated in the graph.
